# Chemical Composition of Essential Oils from Eight Tunisian *Eucalyptus* Species and Their Antifungal and Herbicidal Activities

**DOI:** 10.3390/plants12173068

**Published:** 2023-08-26

**Authors:** Amira Ayed, Flavio Polito, Hedi Mighri, Mouna Souihi, Lucia Caputo, Lamia Hamrouni, Ismail Amri, Filomena Nazzaro, Vincenzo De Feo, Ann M. Hirsch, Yassine Mabrouk

**Affiliations:** 1 Laboratory of Biotechnology and Nuclear Technology, National Center for Nuclear Sciences and Technologies (CNSTN), Sidi Thabet Technopark, Sidi Thabet 2020, Tunisia; ayedamira21@gmail.com (A.A.); souihimouna@gmail.com (M.S.); 2 Higher Institute of Biotechnology of Sidi Thabet (ISBST), University of Manouba, Sidi Thabet 2020, Tunisia; 3 Department of Pharmacy, University of Salerno, Via San Giovanni Paolo II, 132, 84084 Fisciano, Italy; fpolito@unisa.it (F.P.); lcaputo@unisa.it (L.C.); defeo@unisa.it (V.D.F.); 4 Range Ecology Laboratory, Arid Region Institute, University of Gabes, El-Jorf Road Km 22.5, Medenine 4119, Tunisia; mighrih@yahoo.fr; 5 Laboratory of Management and Valorization of Forest Resources, National Institute of Researches on Rural Engineering, Water and Forests, P.B. 10, Ariana 2080, Tunisia; hamrounilam@yahoo.fr; 6 Institute of Food Science, National Research Council of Italy, Via Roma, 64, 83100 Avellino, Italy; filomena.nazzaro@isa.cnr.it; 7 Department of Molecular, Cell, and Developmental Biology, Molecular Biology Institute, University of California, Los Angeles, CA 90095, USA; ahirsch@ucla.edu

**Keywords:** *Eucalyptus*, essential oils, antifungal activity, herbicidal activity

## Abstract

*Eucalyptus* species are known to produce metabolites such as essential oils (EOs) that play an important role in the control of weeds, pests and phytopathogenic fungi. The aims of this study were as follows: (i) to determine the chemical composition of the EOs derived from eight *Eucalyptus* species growing in Tunisia, and (ii) to study their possible antifungal and herbicidal activities. EOs were obtained by hydrodistillation from the dried leaves of eight *Eucalyptus* species, namely, *E. angulosa*, *E. cladocalyx*, *E. diversicolor*, *E. microcoryx*, *E. ovata*, *E. resinifera*, *E. saligna* and *E. sargentii*, and the determination of their composition was achieved by GC and GC-MS. The EOs’ antifungal activities were tested against four *Fusarium* strains, and the EOs’ herbicidal properties were evaluated on the germination and seedling growth of three annual weeds (*Trifolium campestre*, *Lolium rigidum* and *Sinapis arvensis*) and three cultivated crop species (*Lepidium sativum*, *Raphanus sativus* and *Triticum durum*). The EO yields ranged between 0.12 and 1.32%. The most abundant components found were eucalyptol, α-pinene, *p*-cymene, *trans*-pinocarveol, α-terpineol and globulol. All EOs showed significant antifungal activity against the four phytopathogenic *Fusarium* strains. *E. cladocalyx* EO exhibited the highest level of antifungal activity, and the greatest inhibition of seed germination was obtained even at lowest concentrations used. These findings suggested that *E. resinifera*, *E. ovata* and *E. cladocalyx* EOs could have applications in agriculture as possible biopesticides, as *Fusarium* antagonists and as bioherbicides.

## 1. Introduction

The term “pesticide” indicates a wide range of compounds such as insecticides, fungicides, herbicides, rodenticides, molluscicides, nematicides and plant growth regulators [1]. In Tunisia, weeds and phytopathogenic fungi can cause high yield loss, attaining 80% [2,3]. Fungi as plant pathogens cause a variety of plant diseases resulting in losses both in food crop production and after harvest [4]. Weeds have been identified as one of the most aggressive agricultural problems, reducing the quality and yield of crop production [5].

In fact, weeds are the main biotic stresses on crop production; they are in competition with crops for natural resources: water, sunlight, space and nutrients. The available literature suggests the substantial yield and economic losses due to weeds. In Tunisia’s cereal production, weeds continue to be an ongoing problem and are one of the limiting factors for growth [6]. To fight against these pests, farmers use synthetic pesticides with negative, harmful effects on the environment and human health. Herbicides are increasingly found in groundwater and surface water due to their extensive use in agricultural systems. In addition, the intensive use of pesticides allows the constant emergence of resistance by all types of organisms [7]. Therefore, biologically active compounds from plants can be used as a potential source of potential fungal, pest and weed control agents [8,9,10]. 

EOs represent a source of compounds with antifungal, pest and weed control potential to be used as an alternative to fill the role of synthetic products. Consequently, for several years there has been a great deal of interest in plant-derived EOs, rich in active compounds which have very important biological properties, as a source of biopesticides [11,12,13]. This is the case for the species of the *Eucalyptus* genus, which is native to Australia, contains approximately 900 species and is a member of the Myrtaceae family [14]. Since 1957, 117 different species of *Eucalyptus* have been introduced to Tunisia [15]. *Eucalyptus* species are now widely distributed around the world, owing principally to their advantageous wood properties for the paper industries [16]. Historically, only a few species belonging to this genus have been employed to extract EOs, primarily from the leaves, for application mostly in the cosmetic and pharmaceutical industries. *Eucalyptus* leaves are rich in EOs, which consist mainly of monoterpenes and sesquiterpenes as well as other secondary metabolites such as phenols, alkaloids, flavonoids and tannins [17].

As well, *Eucalyptus* extracts have been reported to have herbicidal effects on the seedling growth and germination of many weeds [18,19,20]. Several studies have shown the herbicidal properties of EOs against a wide variety of weeds [19,20,21,22].

Hence, the present study aimed to elucidate the chemical composition of the EOs obtained from the leaves of eight Tunisian *Eucalyptus* species and to investigate their antifungal and, for the first time, phytotoxic effects. The antifungal activity of the EOs was assessed against four plant pathogenic *Fusarium* strains, and their herbicidal effects were tested by evaluating their inhibition of the seed germination and seedling growth of three common weeds and three cultivated species. 

## 2. Results

### 2.1. Yields and Chemical Composition

The average EO yields for the eight *Eucalyptus* species ranged from 0.12% for *E. ovata* to 1.32% for *E. sargentii* (Table 1). The EO yields revealed that there are significant differences (*p* < 0.05) between *Eucalyptus* species; seven groups of nonoverlapping EOs were discovered using the statistical test.

The identified phytochemical constituents of *Eucalyptus* EOs are presented in Table 1 according to their order of elution on the SH-RXI-5MS (RT) column, and their percentages were calculated from the flame ionization detector (FID). 

In the current study, the analyzed EOs showed the presence of 41 components in total, accounting for from 96.01 to 98.62% of the total EOs, and each sample showed a specific composition. The 41 identified compounds were classified into six classes.

Twenty-two oxygenated monoterpenes representing 36.49–90.23% were detected; this class is the most important in terms of the number of compounds detected and also in terms of content. Moreover, the EOs contained six monoterpene hydrocarbons (6.54–49.22%), seven oxygenated sesquiterpenes representing 0.3–24.53% of the total and a single sesquiterpene hydrocarbon represented by aromadendrene. Furthermore, three compounds belonging to the class of phenylpropanoides derivatives (0–5.01%) were detected, and this fraction reached its maximum in *E. diversicolor*. In addition, two nonterpenic compounds (0.34–2.18%) were also described. This variability of the subclasses of compounds reflects a great diversity of production of EOs by *Eucalyptus* also supported by a clear variability in the major components of each subclass.

The EOs belonged to a eucalyprol chemotype, with this component ranging from 20.36 to 66.67%, except for *E. resinifera* EO, which belongs to a *p*-cymene chemotype, and the EOs of *E. diversicolor*, characterized by high amounts of α-terpinyl acetate/α-terpineol. 

Isoborneol (10.54%) and aromandendrene (7.2%) in *E. saligna* EOs; α-pinene which reached 14.49% in *E. ovata* oil and β-pinene (8.85%) only in E. cladocalyx EOs; and trans-pinocarveol in *E. sargentii* EOs were detected. In addition, in *E. ovata* EOs, the viridiflorol, α-eudesmol and β-eudesmol content reached 8.13, 5.58 and 2.04%, respectively. The constituents remain in trace in the other EOs.

Other compounds, detected in amounts greater than 1% in some EOs while being totally absent in others, were γ-terpinene (4.65%), ledol (4.31%), spathulenol (4.10%), pinocarvone (3.42%), guaiol (3.13%), thymol (2.8%), verbenol (2.51%), isopentyl valerate (2.18%), α-phellandrene (1.50%), eicosane (1.47%), trans-pulegol (1.21%) and camphene (1.10%). 

### 2.2. Antifungal Activity

**Figure 1:**
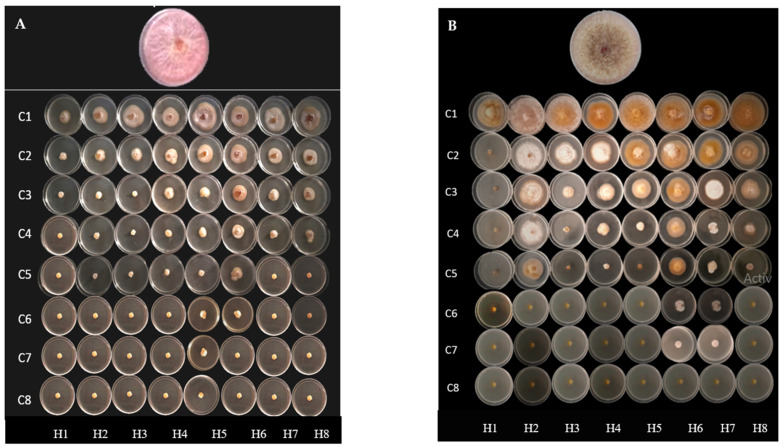

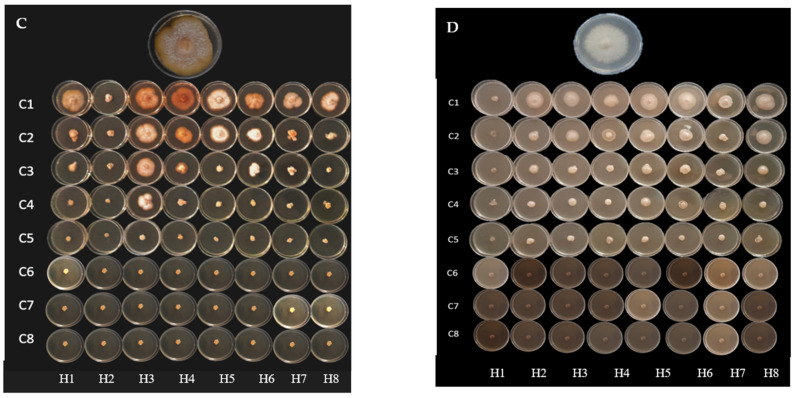
Inhibition of fungal mycelia growth by the EOs at increasing concentrations (C1–C8) from 0 to 8 µL/mL on *F. oxysporum* matthioli (**A**), *F. culmorum* (**B**), *F. oxysporum* solani (**C**) and *F. redolens* (**D**) after seven days of incubation. *E. cladocalyx*: (H1); *E. microcorys*: (H2); *E. resinifera*: (H3); *E. saligna*: (H4); *E. angulosa*: (H5); *E. diversicolor*: (H6); *E. ovata*: (H7); *E. sargentii*: (H8).

The EOs were investigated for their antifungal activity against four phytopathogenic fungal strains. The data, summarized in Table 2, and the results, in Figure 1, show the effects of increasing concentrations of the EOs on the growth of the mycelium of the fungal strains.

The eight EOs inhibited the growth of the four fungal strains in a dose-dependent manner. According to the statistical analysis, there was a significant difference in mycelial growth inhibition between the fungal strains studied. *F. oxysporum* and *F. redolens* were more sensitive to most EOs tested, and a total growth inhibition of these two fungal strains was obtained at doses ranging from 3 to 6 µL/mL and from 1 to 6 µL/mL, respectively. *E. cladocalyx* EO was the most effective, inhibiting mycelial growth up to 100% in all fungal species studied at doses below 3 µL/mL. 

*E. cladocalyx* EO achieved 100% inhibition of mycelial growth for the four pathogens tested with MICs ranging from 1 to 3 µL/mL, the lowest compared to those obtained with the other EOs (Table 3).

The EOs from *E. microcorys*, *E. resinifera* and *E. sargentii* showed MICs varying from 4 to 6 µL/mL. For the EO of *E. saligna*, the MIC was 5 µL/mL for all fungal strains. MICs reached 8 µL/mL for *E. angulosa*, *E. diversicolor* and *E. ovata* EOs.

The EO of E. cladocalyx proved to be the most lethal, with a minimum fungicide concentration (MFC) of 3 µL/mL for the various pathogens. The second most toxic was the EO of *E. sargentii*, with an MFC of 6 µL/mL against three out of four fungal strains tested (Table 2). *E. ovata* EO did not show fungicidal activity on the four pathogens studied at the concentrations tested. Although the mycelium growth of *F. oxysporum* and *F. oxysporum* f. *matthioli* was inhibited at low doses of some EOs, these two strains were more tolerant to the majority of EOs, with MFCs greater than 8 μL/mL. 

In addition, in this study, the antifungal potential of fosetyl-Al (aluminum tris-O-ethylphosphonate), a commercial fungicide, was also evaluated; the main results are presented in Table 4.

Obtained results showed that the fungal species show great differences in their sensitivity to fosetyl-Al. *F. oxysporum* solani was the most sensitive with an MIC of 1 mg/mL, followed by the two strains *F. culmorum* and *F. oxysporum* f. sp. *matthioli* (MIC = 1.5 mg/mL). The *F. redolens* strain was the most resistant, with total inhibition of mycelium growth observed at 3 mg/mL of fosetyl-Al. The effects of fosetyl-Al on the growth of the fungi studied are comparable to those obtained with the EOs extracted from *Eucalyptus* species.

### 2.3. Herbicidal Effects of the EOs on Germination and Seedling Growth of Weeds and Cultivated Crops 

The EOs were assessed for their phytotoxic potential against weeds widely found in Tunisia: *Trifolium campestre* Schreb., *Sinapis arvensis* L. (dicots) and *Lolium rigidum* Gaudich. (monocot); they were also assessed against cultivated crops: *Lepidium sativum* L., *Raphanus sativus* L. (dicots) and *Triticum durum* Desf. (monocot). The results are summarized in Table 5, Table 6 and Table 7.

**Figure 2:**
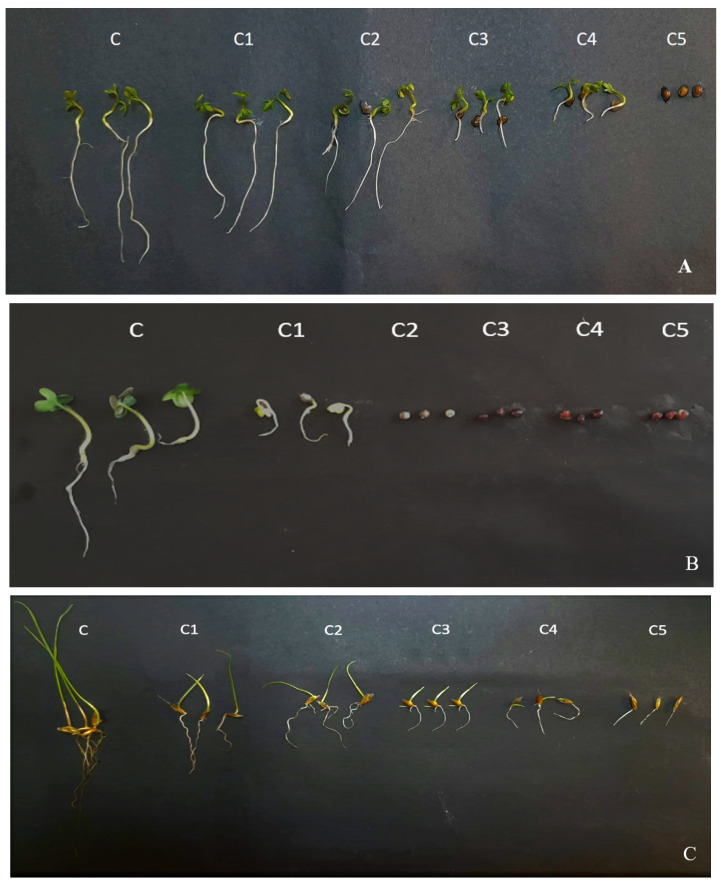
Phytotoxic effects of the EOs on *L. sativum* L. (**A**), *S. arvensis* L. (**B**) and *L. rigidum* Gaudich. (**C**). C: 0 μL/mL; C1: 1 μL/mL; C2: 2 μL/mL; C3: 3 μL/mL; C4: 4 μL/mL; C5: 5 μL/mL.

All EOs strongly reduced the germination and seedling growth of the tested plants. Varying degrees of inhibition were observed depending on the EO, the concentrations and the response of the species, as shown in Figure 2.

The data obtained show that, compared to the other EOs, the *E. cladoclayx* EO is the most powerful inhibitor agent of dicots. In fact, it fully inhibits the germination and seedling growth of these weeds at doses ranging from 1 to 3 μL/mL.

However, *E. saligna* EO showed the most effective results against monocots, completely inhibiting the germination and growth of *L. rigidum* Gaudich. at 2 μL/mL. These EOs showed more efficient results than the synthetic herbicide tested (glyphosate).

On the other hand, weeds have shown different degrees of sensitivity, and *S. arvensis* L., which is regarded as a highly aggressive weed in cereal fields, has proved to be the most sensitive. Indeed, at a low dose (1 μL/mL), three EOs (*E. ovata*, *E. microcorys* and *E. cladocalyx*) among the eight tested severely inhibited the germination and growth of the roots and aerial parts of *S. arvensis* L. without harming the growth of the cultivated crops tested.

## 3. Discussion

The results showed that the EO yield in *E. cladocalyx*, grown in the arboretum of Zerniza in the region of Sejnene in northwest Tunisia, was comparable to those obtained from Algeria (0.49%) and Morocco (0.30%) [23,24], but much lower than those obtained in another Tunisian site (arboretum of Mjez elbal Beja) (5.1 ± 0.4%) [25]. For *E. ovata*, the richness of the EO is the same as in those grown in Algeria [24]. *E. sargentii* showed the highest yield in comparison to other species in our study, but this yield was significantly lower than that obtained from the same species in other regions in Tunisia [26]. The differences between these yields and those reported in the literature could be attributed to many factors, such as the age of the tree, the climatic conditions, the edaphic conditions and the method of extraction [19,25,26].

In total, 41 components were determined, and the highest number (36) were identified in *E. ovata* EO, while only 17 components were identified in *E. angulosa* EO. Eucalyptol was the main component in all EOs except in those from *E. resinifera* and *E. diversicolor*. These results confirm what has been described by authors who reported that the monoterpene eucalyptol was the dominant component for the main species of the genus *Eucalyptus*. Kouki et al. (2022) reported that eucalyptol was the main component in some *Eucalyptus* species growing in Tunisia with percentages between 44.9 and 78.1% [27]. Our analysis showed that the α-pinene content takes the second position in some species such as *E. ovata*, *E. sargenti* and *E. angulosa*, which agrees with several previous works [28,29]. These results were partially in agreement with the data described by an Algerian team showing *E. ovata* EO contents of 51.2 and 7.8% for 1,8-cineole and α-pinene, respectively, whereas for *E. saligna* EO, the major component was α-phellandrene (16.8%) followed by *p*-cyrnene (14.5%) [23].

In Morocco, two studies reported the composition of *E. cladocalyx* EO; the first identified 24 compounds representing about 81.1% of the EO, with α-pinene (23%), *p*-cymene (16.3%) and 1,8-cineole (13.7%) as the most prominent components, followed by β-pinene (6.3%), *trans*-pinocarveol (4.3%) and α-terpineol (4%) [24]. In the second study, Fouad et al. (2015) identified 29 different components representing 79% of the total oil [30]. The major components detected were spathulenol (21.6%) and 1,8-cineole (20.5%), followed by *p*-cymene (15.1%). In Tunisia, the work of Ben Hassine et al. (2010) described only six compounds in the EO of *E. cladocalyx*, whose major compounds were 1,8-cineole (71.19%) and α-thujene (5.53%) [31]. Ameur et al. (2021) identified 23 compounds including globulol (12.7%). In our study, the EO of *E. cladocalyx* was rich in eucalyptol (44.68%) as the major component, followed by β-pinene (8.85%) [25].

Regarding the EOs of *E. diversicolor*, the results described by Elaissi et al. (2011) partly agree with our conclusions concerning the presence of *p*-cymene [32]. On the other hand, a Moroccan EO was characterized by the high content of 1,8-cineole and the absence of *p*-cymene [24].

In vitro tests revealed that all EOs inhibited fungal growth in a dose-dependent way, which agrees with many reports in the literature on the concentration-dependent antifungal activity of essential oil [33,34].

In this study, the EOs tested elicited antifungal activity for the four phytopathogenic fungal strains (*F. oxysporum*, *F. culmorum*, *F. oxysporum* f. *matthioli* and *F. redolens*), and this mycelial growth inhibition was variable depending on the concentration and nature of the essential oil. These findings agree with those of previous studies that evaluated the antifungal activity against various phytopathogenic fungi of the EOs of some *Eucalyptus* species with different chemical profiles. Recently, we reported that some EOs inhibited the growth of eight phytopathogenic fungi [35]. *E. citriodora* Hook. completely inhibited the growth of seven strains belonging to the genus *Fusarium* at a concentration of 4 μL/mL. Another study showed that EOs from *E. oleosa* F. Muell. ex Miq. inhibited fungal growth with MICs of 6 µL/mL for five strains of *Fusarium* [36].

In our work, the EO of *E. cladocalyx* showed fungicidal activity against all four *Fusarium* strains tested at low dose, whereas the other EOs were fungicidal only against one or two of the four fungal strains. The EOs of *E. ovata* at the four concentrations studied did not exhibit fungicidal activity. Consequently, higher concentrations are needed to obtain the MFC. In vitro tests revealed that all EOs inhibited fungal growth in a dose-dependent way, which agrees with many reports in the literature on the concentration-dependent antifungal activity of essential oil [35,36].

In particular, the EO of *E. cladocalyx* showed a remarkable antifungal activity against phytopathogenic fungi that have a devastating effect on several species cultivated in Tunisia. This EO is characterized by the abundance of some compounds such as eucalyptol, pinene, *trans*-pinocarveol and α-terpineol, which may explain its antifungal power. Several previous works have shown the involvement of 1,8-cineole in the inhibition of phytopathogenic fungi [37].

Based on the literature and according to Table 1, several compounds detected in the oils studied are known to have antifungal potential. According to Kim et al. (2012), 16 pure compounds of EOs were tested for their antifungal potential; among these compounds were α-pinene, 1,8-cineole and *p*-cymene, which constitute the major compounds of *Eucalyptus* tested in our present study. These compounds showed significant antifungal potential. This could explain the results of the current study [38]. Similarly, α-pinene, a major compound for the oils studied (0.8–14.49%), is a monoterpene that has been demonstrated to inhibit respiration and ion transport and to act on cell integrity by increasing membrane permeability [39,40,41]. In addition, 1,8-cineole, an oxygenated monoterpene and major compound in this study (3.22–66.67%), has been described for its antifungal potential against phytopathogenic fungi [42,43,44]. Similarly, according to Kim et al. (2018), 1,8-cineole showed a significant inhibition of growth and the production of aflatoxin B1 and aflatoxin B2 of several fungi strains, and these antifungal properties were explained by a dramatic downregulation of 1, 8-cineole on the expression of afl E and afl L [45]. This can explain the activities observed in this present study without neglecting the role of other compounds, thus without neglecting the interactions of synergism and antagonism. A research study reported that some components of *Eucalyptus* EOs such as α-terpineol, terpinolene, and 1,8-cineole are fungitoxic against phytopathogenic fungi [46]. Regarding the antifungal mechanism, previous work has reported that the apolar terpenes can penetrate the lipid bilayer of the fungal membrane using their apolar properties. Hence, terpenes induce fungal membrane disruption by increasing the membrane’s permeability [47].

Our report showed that *Eucalyptus* EOs exhibit herbicidal activity for all species tested, with more noticeable effects on weeds. In agreement with our findings, previous works showed the herbicidal effects of *Eucalyptus* EOs against several weeds and crop plants [48,49,50]. Thus, some components of *Eucalyptus* EOs are known for their phytotoxic effects and can be used as natural herbicides. Nevertheless, the phytotoxic activity of *Eucalyptus* EOs may affect some crops [51]. It is therefore important to develop research to select the EOs with maximum herbicidal activity against weeds while minimizing negative impacts on crop growth. Seven of the eight EOs studied contain appreciable percentages of eucalyptol correlated with significant herbicidal activity. The high levels of eucalyptol, a monoterpene with phytotoxic properties, may partly explain the results obtained [52,53,54]. The greatest inhibition was obtained using the EO of *E. cladocalyx*, which has appreciable levels of 1,8-cineole, α-pinene, β-pinene, *trans*-pinocarveol and α-terpineol. These findings suggest that 1,8-cineole combined with other terpenes may provide significant phytotoxic effects [29,55,56]. According to the chemical composition of the oils of the eight *Eucalyptus* trees studied (Table 1), we can notice the presence of several compounds known for their herbicidal activities, such as 1,8-cineole, α-pinene and *p*-cymene, detected as major compounds of the tested oils [54,55,56]. These findings confirm a synergy between the various constituents of EOs for the observed phytotoxic effects.

Although some studies have tried to explain the mechanisms of action of the EOs on germination and inhibition of the growth of seedlings, these modes of action remain unclear. Previous reports have suggested a number of effects and hypotheses; the majority of researchers working on this topic agree that EOs have phytotoxic effects that can cause anatomical and physiological changes in plant seedlings leading to accumulation of lipid globules in the cytoplasm, reduction of certain organelles such as mitochondria, inhibition of DNA synthesis or disruption of membranes surrounding mitochondria and nuclei [57,58]. In this way, the development of natural pesticides and herbicides would help to reduce the negative impact of chemicals, such as the development of resistance in pathogens and parasites and resistant weeds. To this end, biopesticides and bioherbicides can be effective, selective, biodegradable and less harmful to the environment and human health. This study reports the potential antifungal and herbicidal effects of the essential oils from eight *Eucalyptus* species.

## 4. Materials and Methods

### 4.1. Plant Material

Leaf samples were collected from 8 *Eucalyptus* species growing in different regions of Tunisia under different climatic conditions. Voucher specimens of *Eucalyptus* species were identified by Professor Hamrouni Lamia, and a voucher specimen with an assigned code for each sample was deposited at the Laboratory of Genetic and Forest Ecology of the National Research Institute of Rural Engineering, Water and Forests, Tunisia. The relative data for each sample are summarized in Table 8

The leaves were taken from three or more separate trees of each species; the obtained samples were stored in a dry area for two weeks and mixed for homogenization.

### 4.2. Essential Oils Extraction

The EOs were extracted by hydrodistillation of dried *Eucalyptus* leaves for three hours using a Clevenger-type device. Then, EOs were collected, dried and stored in dark vials at 4 °C in a refrigerator until analysis and bioassays. After recovery of the EOs, the yield was expressed as a percentage (%) and calculated according to the following formula:

Essential oil yield (%) = (mEO/mVM) × 100

where mEO is the mass of recovered essential oils in grams (g) and mVM is the dry vegetable material mass in grams (g).

### 4.3. Analysis of the EOs

The analysis was carried out using a GC/MS-QP2010 Ultra (Shimadzu, Kyoto, Japan) and a capillary column (30 m × 0.25 mm × 0.25 µm film thickness) SH-RXI-5MS (5% diphenyl/95% dimethyl polysiloxane). The oven temperature was programmed from 50 to 250 °C at 7 °C/min; injector temperature: 250 °C; carrier gas: helium (1.0 mL/min); sample automatically injected: 0.01 mL.

The mass spectrometer conditions were as follows: ionization voltage, 70 eV; ion source temperature, 200 °C; electron ionization mass spectra were acquired over the mass range 50–550 m/z. The Kovats indices were calculated related to a series of *n* alkanes (C8–C30). The components of each oil were identified by their Kovats indices and comparison of their mass spectra with those in the literature libraries [59] and by co-injection of standards if available. The quantitative analysis of each oil component (%) was carried out by peak area normalization measurement.

### 4.4. Antifungal Activity

Four phytopathogenic fungal strains (*Fusarium oxysporum solani*, *F. oxysporum* f. sp. *matthioli*, *F. culmorum* and *F. redolens*) were used in this study. The antifungal effect was evaluated in vitro using the agar dilution method [60]. The EOs were diluted in a solution (0.1%) of Tween 20 and then mixed in PDA medium to obtain the desired concentration. A 5 mm disc, cut at the periphery of each fresh fungal culture, was placed in the center of the PDA plate and incubated for 7 days at 24 °C in the dark. The experiments were carried out in triplicate. Tween 20-treated PDA plates (0.1%) were used as a negative control. Fosetyl-Al (aluminum tris-O-ethylphosphonate) was used as drug positive control in wettable powder (AlietteR, Bayer CropScience Ltd, India). The effect of this compound on linear mycelial growth was measured by adding fosetyl-Al to the Tween 20-treated PDA plates at different concentrations (0.2, 0.5, 1, 1.5, 2 and 3 mg/mL).

The following formula was used to determine the growth inhibition as the percentage of radial growth inhibition compared to the control.

Growth inhibition % = (C − T)/C × 100

where C is the mean of three replicates of the controls’ hyphal extension measurement (mm) and T is the mean of three replicates of the hyphal extension (mm) of plates treated with EOs or fosetyl-Al.

The minimum inhibitory concentration (MIC) is the lowest dose that causes complete inhibition of fungal growth. The inhibited fungal discs (MICs) were transferred into new plates containing PDA (without EOs or fosetyl-Al), and their growth was observed to establish the minimum fungicide concentrations (MFC). After 3 days of incubation, MFC was obtained as the lowest MIC at which no growth was observed [60].

### 4.5. Effect of Eucalyptus EOs of Seed Germination and Seedling Growth

Mature seeds of weeds (*Trifolium campestre* Schreb., *Lolium rigidum* Gaudich. and *Sinapis arvensis* L.) and cultivated crops (*Lepidium sativum* L., *Raphanus sativus* L. and *Triticum durum* Desf.) were harvested from mother Tunisian crop fields in July 2019 and were used in phytotoxic tests. In order to prevent potential inhibition from toxins in microorganisms, the seeds were surface-sterilized for 20 min with 5% sodium hypochlorite; then, they were rinsed with sterile distilled water.

To study herbicidal potential, the EOs were dissolved in Tween–water solution (1%; *v*/*v*). Emulsions of 6 mL of the appropriate EO solution were transferred to Petri dishes containing a double-layer filter paper (Whatman No. 1) to obtain final different treatment concentrations (1–5 μL/mL). Then, 10 seeds were placed and spread evenly over the filter paper [61]. Subsequently, the Petri dishes were closed and taped to prevent the escape of volatile EOs. The trials were conducted using a fully randomized model with three replicates and were repeated three times, including the controls. Glyphosate was used as a reference herbicide.

The cultures were maintained for seven days in controlled conditions in a growth chamber, with 16/8 h photoperiod (day/night) at a temperature of 25 °C and 70% relative humidity. Afterwards, the number of germinated seeds and the roots and shoots lengths were measured.

### 4.6. Statistical Analysis

Through the SPSS 26 software package, data collected from EOs analysis and antifungal, germination, seedling and root growth testing were subjected to ANOVA (unidirectional analysis of variance). Differences between the means were evaluated by the Student–Newman–Keuls test at the *p* values of 0.05.

## 5. Conclusions

This study consists of a contribution to the development of forest bioresources through the screening of the active molecules and biological activities of *Eucalyptus* species. The richness in essential oils of the eight studied species of *Eucalyptus* in this study and, likewise, a diversity of their chemical compositions has been described. The yield is variable, and the oils studied showed a specific richness in hydrocarbon and oxygenated monoterpenes; likewise, the main major compounds were 1,8-cineole, α-pinene, *p*-cymene, *trans*-pinocarveol, α-terpineol and α-terpinyl acetate. Similarly, the variability of the chemical composition was correlated with the biological activities. In fact, *E. cladocalyx* EO was found to be most active against the fungal pathogens tested and reduced seed germination rate and inhibited seedlings, suggesting that these EOs could be used in the formulation of alternative biopesticides. However, further studies are needed to determine the applicability, safety and effect on soil microorganisms of these potential fungicidal, herbicidal and pesticidal agents.

## Figures and Tables

**Table 1:** Chemical composition of the EOs of *Eucalyptus* cladocalyx F. Muell. (A), *E. microcorys* F. Muell. (B), *E. resinifera* Sm. (C), *E. saligna* Sm. (D), *E. angulosa* Schauer (E), *E. diversicolor* F. Muell. (F), *E. ovata* Labill. (G) and *E. sargentii* Maiden (H).

Compounds	RT (min)	Class	A	B	C	D	E	F	G	H	Identification
α-pinene	6.930	MH	6.69	5.6	3.04	2.5	9.19	0.8	14.49	9.48	MS, LRI, IN
Camphene	7.214	MH	-	0.94	0.63	1.1	-	-	0.21	-	MS, LRI
β-pinene	7.884	MH	8.85	-	-	-	-	-	0.59	-	MS, LRI, IN
α-phellandrene	8.552	MH	-	-	0.56	0.61	-	-	1.5	0.46	MS, LRI, IN
*p*-cymene	8.713	MH	-		44.79	15.27	-	20.88	1.9	-	MS, LRI, IN
Eucalyptol	9.193	OM	44.68	60.5	6.36	20.36	66.67	3.22	26.3	55.93	MS, LRI, IN
γ-terpinene	9.862	MH	-	-	0.2	-	-	4.65	0.58	-	MS, LRI, IN
*cis*-linalool oxide	10.190	OM	-	-	0.35	0.82	-	0.56	0.22	-	MS, LRI
Verbenol	10.580	OM	0.37	0.38	0.56	0.57	-	0.47	0.37	0.23	MS, LRI
Isopentyl valerate	10.792	NT	0.34	1.22	0.23	0.42	0.32	0.28	2.18	0.87	MS, LRI
Fenchol	10.987	OM	0.35	2.97	1.91	3.72	0.4	0.89	0.6	0.2	MS, LRI
α-campholenal	11.230	OM	1.26	0.23	-	0.45	-	-	0.59	-	MS, LRI
*trans*-pinocarveol	11.878	OM	6.63	7.71	7.39	4.35	8.91	0.47	4.42	17.54	MS, LRI
Verbenol	12.284	OM	1.57	-	-	0.2	-	-	0.7	-	MS, LRI, IN
Pinocarvone	12.381	OM	0.2	6.72	3.42	1.64	1.88	-	2.51	-	MS, LRI
Isoborneol	12.478	OM	3.34	6.23	4.81	10.54	0.59	0.68	1.33	5.53	MS, LRI
Terpinen-4-ol	12.700	OM	1.1	0.34	1.08	0.65	0.97	4.03	0.97	-	MS, LRI, IN
α-terpineol	12.915	OM	8.6	3.41	6.46	7.44	1.68	17.72	3.85	1.02	MS, LRI, IN
Myrtenol	13.159	OM	0.86	0.34	0.43	0.24	-	-	0.35	0.43	MS, LRI, IN
*trans*-pulegol	13.277	OM	-	-	0.24	-	-	1.21	0.48	-	MS, LRI, IN
Verbenone	13.393	OM	1.56	-	-	-	-	-	0.2	-	MS, LRI
*cis*-carveol	13.624	OM	0.78	0.26	0.37	0.42	-	0.67	0.29	-	MS, LRI
Verbenol	13.823	OM	0.73	0.88	2.51	1.14	0.33	3.13	0.39	0.67	MS, LRI
Cumaldehyde	14.053	OM	2.06	0.26	0.31	-	0.2	1.12	0.35	0.32	MS, LRI
Piperetone	14.398	OM	0.29	-	0.29	-	-	0.7	1.61	-	MS, LRI
Phellandral	15.176	OM	0.32	-	-	-	-	-	-	-	MS, LRI
*p*-cymen-7-ol	15.248	OM	0.48	-	-	0.28	-	-	-	-	MS, LRI
Thymol	15.316	PP	-	-	1.21	-	-	2.8	-	-	MS, LRI, IN
Carvacrol	15.419	PP	0.37	-	-	0.4	-	1.07	1.02	-	MS, LRI, IN
α-terpinyl acetate	16.882	OM	-	-	-	0.34	-	27.44	0.33	-	MS, LRI
Linalyl isobutyrate	16.950	OM	-	-	-	0.88	-	-	0.21	-	MS, LRI
Aromandendrene	18.281	SH	-	-	0.38	7.2	-	-	2.37	-	MS, LRI
Isoamyl phenylacetate	19.098	PP	0.22	0.33	-	0.21	-	1.14	1.11	0.4	MS, LRI
Spathulenol	20.188	OS	1.35	0.3	3.1	4.1	1.69	0.36	-	0.88	MS, LRI, IN
Caryophyllene oxide	20.925	OS	-	-	-	-	-	-	2.01	-	MS, LRI
Viridiflorol	21.113	OS	3.44	-	2.04	2.63	1.45	0.32	8.13	0.53	MS, LRI
Ledol	21.225	OS	0.77	-	2.39	3.83	0.81	-	4.31	0.36	MS, LRI
Guaiol	21.687	OS	-	-	-	3.13	-	-	2.46	0.22	MS, LRI
β-eudesmol	21.758	OS	-	-	1.05	0.28	0.78	-	2.04	1.92	MS, LRI
α-eudesmol	22.278	OS	-	-	0.69	0.29	0.63	0.23	5.58	-	MS, LRI
Eicosane	31.482	NT	-	-	0.27	-	0.47	1.47	-	0.21	MS, LRI
Total identification			97.21	98.62	97.07	96.01	96.97	96.31	96.55	97.2	
* Yield (*w*/*w* %)			0.2 ^b^	0.56 ^c^	0.81 ^e^	0.64 ^d^	1 ^f^	0.59 ^c,d^	0.12 ^a^	1.32 ^g^	
Monoterpene hydrocarbons		(MH)%	15.54	6.54	49.22	19.48	9.19	26.33	19.27	9.94	
Oxygentated monoterpenes		(OS)%	75.18	90.23	36.49	54.04	81.63	62.31	46.07	81.87	
Sesquiterpene hydrocarbons		(SH)%	-	-	0.38	7.2	-	-	2.37	-	
Oxygentated sesquiterpenes		(OS)%	5.56	0.3	9.27	14.26	5.36	0.91	24.53	3.91	
Phenylpropanoids		(PP)%	0.59	0.33	1.21	0.61	0	5.01	2.13	0.4	
Non-terpene derivatives		(NT)%	0.34	1.22	0.5	0.42	0.79	1.75	2.18	1.08	

Components are listed in their order of elution from an SH-RXI-5MS column, and their percentages were calculated from a flame ionization detector (FID); RT: retention time; -: not detected; LRI: linear retention index; MS: mass spectrometry; IN: co-injection with authentic compounds; *: means of EOs yield of *Eucalyptus* species followed by the same letter are not significantly different according to multivariate analysis ANOVA (*p* ≤ 0.05). Data are the mean of three.

**Table 2:** Inhibitory effects of essential oils on the growth percentage of four fungal strains in an agar diffusion plate assay.

*Eucalyptus* Species	Doses (μL/mL)	*F. oxysporum* Solani	*F. culmorum*	*F. oxysporum* f. sp. *matthioli*	*F. redolens*
*E. cladocalyx*	0	0 ± 0 ^A,a^	0 ± 0 ^A,a^	0 ± 0 ^A,a^	0 ± 0 ^A,a^
	1	78.84 ± 0.64 ^B,b^	58.88 ± 0.64 ^B,c^	85.55 ± 0.32 ^B,b^	100 ± 0 ^B,a^
	2	90 ± 0 ^C,c^	100 ± 0 ^C,a^	94.44 ± 0 ^C,b^	100 ± 0 ^B,a^
	3	100 ± 0 ^D,a^	100 ± 0 ^C,a^	100 ± 0 ^D,a^	100 ± 0 ^B,a^
	4	100 ± 0 ^D,a^	100 ± 0 ^C,a^	100 ± 0 ^D,a^	100 ± 0 ^B,a^
	5	100 ± 0 ^D,a^	100 ± 0 ^C,a^	100 ± 0 ^D,a^	100 ± 0 ^B,a^
	6	100 ± 0 ^D,a^	100 ± 0 ^C,a^	100 ± 0 ^D,a^	100 ± 0 ^B,a^
	7	100 ± 0 ^D,a^	100 ± 0 ^C,a^	100 ± 0 ^D,a^	100 ± 0 ^B,a^
	8	100 ± 0 ^D,a^	100 ± 0 ^C,a^	100 ± 0 ^D,a^	100 ± 0 ^B,a^
*E. microcorys*	0	0 ± 0 ^A,a^	0 ± 0 ^A,a^	0 ± 0 ^A,a^	0 ± 0 ^A,a^
	1	74.44 ± 0 ^B,a^	44.44 ± 0 ^B,c^	76.66 ± 0.32 ^B,a^	67.18 ± 0.25 ^B,b^
	2	92.22 ± 0.64 ^C,a^	63.32 ± 0.32 ^C,c^	81.10 ± 0.32 ^C,b^	81.53 ± 0 ^C,b^
	3	93.33 ± 0.02 ^D,a^	74.44 ± 0.64 ^E,c^	94.44 ± 0 ^D,a^	84.61 ± 0.01 ^D,b^
	4	100 ± 0 ^E,a^	88.88 ± 0 ^F,c^	96.66 ± 0 ^E,b^	90.25 ± 0.51 ^E,d^
	5	100 ± 0 ^E,a^	94.44 ± 0 ^G,b^	97.77 ± 0 ^F,b^	95.38 ± 0 ^F,b^
	6	100 ± 0 ^E,a^	100 ± 0 ^G,a^	100 ± 0 ^G,a^	100 ± 0 ^G,a^
	7	100 ± 0 ^E,a^	100 ± 0 ^G,a^	100 ± 0 ^G,a^	100 ± 0 ^G,a^
	8	100 ± 0 ^E,a^	100 ± 0 ^G,a^	100 ± 0 ^G,a^	100 ± 0 ^G,a^
*E. resinifera*	0	0 ± 0 ^A,a^	0 ± 0 ^A,a^	0 ± 0 ^A,a^	0 ± 0 ^A,a^
	1	70 ± 1.92 ^B,d^	44.44 ± 0.64 ^B,c^	59.99 ± 0.32 ^B,f^	65.38 ± 0.44 ^B,f^
	2	84.44 ± 0.25 ^C,a^	68.88 ± 0.32 ^C,c^	83.33 ± 0 ^C,a^	78.72 ± 0.25 ^C,b^
	3	87.77 ± 0.64 ^D,b^	88.88 ± 0.67 ^D,b^	97.77 ± 0.57 ^D,a^	86.15 ± 0 ^D,b^
	4	100 ± 0 ^E,a^	91.11 ± 0 ^E,b^	100 ± 0 ^E,a^	89.23 ± 0.01 ^E,e^
	5	100 ± 0 ^E,a^	100 ± 0 ^F,a^	100 ± 0 ^E,a^	100 ± 0 ^F,a^
	6	100 ± 0 ^E,a^	100 ± 0 ^F,a^	100 ± 0 ^E,a^	100 ± 0 ^F,a^
	7	100 ± 0 ^E,a^	100 ± 0 ^F,a^	100 ± 0 ^E,a^	100 ± 0 ^F,a^
	8	100 ± 0 ^E,a^	100 ± 0 ^F,a^	100 ± 0 ^E,a^	100 ± 0 ^F,a^
*E. saligna*	0	0 ± 0 ^A,a^	0 ± 0 ^A,a^	0 ± 0 ^A,a^	0 ± 0 ^A,a^
	1	64.44 ± 0 ^B,d^	59.66 ± 0.11 ^B,a^	64.44 ± 0 ^B,d^	75.38 ± 0.04 ^B,c^
	2	71.11 ± 0 ^C,b^	72.22 ± 0.28 ^C,b^	81.55 ± 0.19 ^C,a^	78.46 ± 0.02 ^C,b^
	3	92.22 ± 0.75 ^D,e^	77.77 ± 0.51 ^D,c^	83.33 ± 0.32 ^D,f^	90.76 ± 0 ^D,b^
	4	97.77 ± 0 ^E,b^	81.11 ± 0 ^E,e^	96.66 ± 0.19 ^E,b^	92.3 ± 0 ^E,b^
	5	100 ± 0 ^F,a^	100 ± 0 ^F,a^	100 ± 0 ^F,a^	100 ± 0 ^F,a^
	6	100 ± 0 ^F,a^	100 ± 0 ^F,a^	100 ± 0 ^F,a^	100 ± 0 ^F,a^
	7	100 ± 0 ^F,a^	100 ± 0 ^F,a^	100 ± 0 ^F,a^	100 ± 0 ^F,a^
	8	100 ± 0 ^F,a^	100 ± 0 ^F,a^	100 ± 0 ^F,a^	100 ± 0 ^F,a^
*E. angulosa*	0	0 ± 0 ^A,a^	0 ± 0 ^A,a^	0 ± 0 ^A,a^	0 ± 0 ^A,a^
	1	66.66 ± 0.57 ^B,d,e^	44.44 ± 0.25 ^B,c^	58.88 ± 0.32 ^B,g^	66.15 ± 0.0 ^B,f^
	2	79.99 ± 0.64 ^C,e^	61.11 ± 0 ^C,a^	80 ± 0.57 ^C,e^	76.92 ± 0 ^C,d^
	3	83.33 ± 0 ^D,b^	73.33 ± 0 ^D,c^	87.77 ± 0 ^D,a^	83.07 ± 0.04 ^D,b^
	4	91.11 ± 0.64 ^E,a^	83.33 ± 1.15 ^E,b^	90 ± 1.15 ^E,a^	86.61 ± 0 ^E,b^
	5	100 ± 0 ^F,a^	100 ± 0 ^F,a^	94.44 ± 0.06 ^F,c^	91.79 ± 0.51 ^F,c^
	6	100 ± 0 ^F,a^	100 ± 0 ^F,a^	95.55 ± 0 ^F,b^	100 ± 0 ^G,a^
	7	100 ± 0 ^F,a^	100 ± 0 ^F,a^	97.77 ± 0 ^G,b^	100 ± 0 ^G,a^
	8	100 ± 0 ^F,a^	100 ± 0 ^F,a^	98.88 ± 0 ^G,b^	100 ± 0 ^G,a^
*E. diversicolor*	0	0 ± 0 ^A,a^	0 ± 0 ^A,a^	0 ± 0 ^A,a^	0 ± 0 ^A,a^
	1	66.66 ± 0.64 ^B,d,e^	44.44 ± 0.64 ^B,c^	64.44 ± 0.02 ^B,d^	63.84 ± 0.44 ^B,g^
	2	71.11 ± 0.64 ^C,f^	56.66 ± 0.64 ^C,g^	75.55 ± 0.02 ^C,f^	75.38 ± 0.01 ^C,e^
	3	74.44 ± 0 ^D,h^	72.22 ± 0 ^D,e^	81.11 ± 0.32 ^D,g^	84.61 ± 0 ^D,e^
	4	79.25 ± 0.98 ^E,d^	77.77 ± 0.44 ^E,a^	87.77 ± 0.57 ^E,e^	88.46 ± 0.02 ^E,f^
	5	95.55 ± 0 ^F,b^	81.11 ± 0 ^F,d^	90 ± 0 ^F,c^	100 ± 0 ^F,a^
	6	100 ± 0 ^G,a^	83.33 ± 0 ^G,c^	96.66 ± 0 ^G,b^	100 ± 0 ^F,a^
	7	100 ± 0 ^G,a^	91.11 ± 0.64 ^H,c^	100 ± 0 ^H,a^	100 ± 0 ^F,a^
	8	100 ± 0 ^G,a^	100 ± 0 ^I,a^	100 ± 0 ^H,a^	100 ± 0 ^F,a^
*E. ovata*	0	0 ± 0 ^A,a^	0 ± 0 ^A,a^	0 ± 0 ^A,a^	0 ± 0 ^A,a^
	1	92.22 ± 0.64 ^B,a^	47.7 ± 0 ^B,d^	68.88 ± 0.32 ^B,c^	76.40 ± 0.51 ^B,b^
	2	95.55 ± 0 ^C,a^	58.88 ± 0 ^C,d^	80 ± 0 ^C,c^	82.04 ± 0.25 ^C,b^
	3	97.77 ± 0 ^D,a^	72.22 ± 0.32 ^D,c^	88.88 ± 0 ^D,b^	89.23 ± 0 ^D,b^
	4	98.88 ± 0 ^E,ab^	83.33 ± 0 ^E,c^	96.33 ± 0.11 ^E,b^	92.3 ± 1.73 ^E,b^
	5	100 ± 0 ^F,a^	88.88 ± 0 ^F,c^	100 ± 0 ^F,a^	100 ± 0 ^F,a^
	6	100 ± 0 ^F,a^	91.11 ± 0 ^G,b^	100 ± 0 ^F,a^	100 ± 0 ^F,a^
	7	100 ± 0 ^F,a^	94.44 ± 0 ^H,b^	100 ± 0 ^F,a^	100 ± 0 ^F,a^
	8	100 ± 0 ^F,a^	100 ± 0 ^I,a^	100 ± 0 ^F,a^	100 ± 0 ^F,a^
*E. sargentii*	0	0 ± 0 ^A,a^	0 ± 0 ^A,a^	0 ± 0 ^A,a^	0 ± 0 ^A,a^
	1	66.66 ± 0.64 ^B,d,e^	44.44 ± 0 ^B,c^	61.11 ± 0.32 ^B,e^	70.76 ± 0.01 ^B,d^
	2	88.88 ± 0.57 ^C,c^	63.33 ± 1.15 ^C,d^	78.88 ± 0.32 ^C,e^	72.3 ± 0.44 ^C,f^
	3	94.44 ± 0 ^D,c^	74.44 ± 0.32 ^D,d^	87.77 ± 0.32 ^D,e^	75.12 ± 0.25 ^D,g^
	4	100 ± 0 ^E,a^	88.88 ± 0 ^E,d^	94.44 ± 0 ^E,b^	91.53 ± 0.01 ^E,c^
	5	100 ± 0 ^E,a^	94.44 ± 0.57 ^F,b^	100 ± 0 ^F,a^	100 ± 0 ^F,a^
	6	100 ± 0 ^E,a^	100 ± 0 ^G,a^	100 ± 0 ^F,a^	100 ± 0 ^F,a^
	7	100 ± 0 ^E,a^	100 ± 0 ^G,a^	100 ± 0 ^F,a^	100 ± 0 ^F,a^
	8	100 ± 0 ^E,a^	100 ± 0 ^G,a^	100 ± 0 ^F,a^	100 ± 0 ^F,a^

Means with different capital letters in the same column and for the same tested oil compare the difference between doses, and means with different lowercase letters in the same line and for the same dose compare the different sensitivities between fungi strains according to the Student–Newman–Keuls test at (*p* ≤ 0.05).

**Table 3:** Fungistatic and fungicidal activities of *Eucalyptus* EOs on mycelia growth of four Fusarium strains.

	*F. oxysporum* Solani		*F. culmorum*		*F. oxysporum* f. *matthioli*		*F. redolens*	
*Eucalyptus* species	MIC (μL/mL)	MFC (μL/mL)	MIC (μL/mL)	MFC (μL/mL)	MIC (μL/mL)	MFC (μL/mL)	MIC (μL/mL)	MFC (μL/mL)
***E. cladocalyx***	3	3	2	3	3	3	1	3
***E. microcorys***	4	>8	6	8	6	>8	6	7
***E. resinifera***	4	>8	5	8	4	8	5	8
***E. saligna***	5	>8	5	7	5	>8	5	8
***E. angulosa***	5	>8	5	7	8	6	6	7
***E. diversicolor***	6	>8	8	8	7	>8	5	>8
***E. ovata***	5	>8	8	>8	5	>8	5	>8
***E. sargentii***	4	>8	6	6	5	6	5	6

MIC: minimum inhibitory concentration; MFC: minimum fungicide concentration.

**Table 4:** Effects of fosetyl-Al on mycelial growth of four fungal strains in an agar diffusion plate assay.

	Percentage Inhibition of Radial Growth								
Fosetyl-Al Doses (mg/mL)	0	0.25	0.5	1	1.5	2	3	MIC (mg/mL)	MFC (mg/mL)
**Fungal strains**									
***F. oxysporum*** **solani**	0 ± 0 ^A^	56.64 ± 1.37 ^B^	90.02 ± 0.86 ^C^	100 ± 0 ^D^	100 ± 0 ^D^	100 ± 0 ^D^	100 ± 0 ^D^	1	>3
***F. culmorum***	0 ± 0 ^A^	42.05 ± 0.64 ^B^	82.24 ± 0.43 ^C^	94.86 ± 0.48 ^D^	100 ± 0 ^E^	100 ± 0 ^E^	100 ± 0 ^E^	1.5	3
***F. oxysporum*** **f. sp. *matthioli***	0 ± 0 ^A^	37.78 ± 1.11 ^B^	55.91 ± 0.68 ^C^	91.59 ± 0.75 ^D^	100 ± 0 ^E^	100 ± 0 ^E^	100 ± 0 ^E^	1.5	>3
***F. redolens***	0 ± 0 ^A^	39.02 ± 1.40 ^B^	53.66 ± 0 ^C^	95.12 ± 0 ^D^	96.26 ± 0.08 ^D,E^	97.56 ± 0 ^E^	100 ± 0 ^F^	3	>3

Means with different capital letters in the same line compare the difference between doses according to the Student–Newman–Keuls test at (*p* ≤ 0.05); MIC: minimum inhibitory concentration; MFC: minimum fungicide concentration.

**Table 5:** Inhibitory effect of the *Eucalyptus* EOs on germination percentage of weeds and cultivated crops.

Tested Plants	Doses (μL/mL)	*E. cladocalyx*	*E. microcorys*	*E. resinifera*	*E. saligna*	*E. angulosa*	*E. diversicolor*	*E. ovata*	*E. sargentii*	Glyphosate
*Trifolium campestre* Schreb.	0	100 ± 0 ^A,a^	100 ± 0 ^A,a^	100 ± 0 ^A,a^	100 ± 0 ^A,a^	100 ± 0 ^A,a^	100 ± 0 ^A,a^	100 ± 0 ^A,a^	100 ± 0 ^A,a^	100 ± 0 ^A,a^
	1	66.7 ± 3.3 ^B,b^	83.3 ± 3.3 ^B,a^	83.3 ± 3.3 ^B,a^	70 ± 0 ^B,b^	90 ± 0 ^B,a^	86.7 ± 3.3 ^B,a^	90 ± 0 ^B,a^	90 ± 0 ^B,a^	83.3 ± 3.3 ^B,a^
	2	33.3 ± 3.3 ^C,d^	56.7 ± 3.3 ^C,c^	70 ± 0 ^C,b^	33.3 ± 3.3 ^C,d^	80 ± 0 ^C,a,b^	76.7 ± 3.3 ^C,a,b^	80 ± 0 ^C,a,b^	83.3 ± 3.3 ^C,a^	73.3 ± 3.3 ^C,a,b^
	3	0 ± 0 ^D,f^	10 ± 0 ^D,e^	50 ± 0 ^D,c^	16.7 ± 3.3 ^D,d^	60 ± 0 ^D,b^	70 ± 0 ^C,a^	70 ± 0 ^D,a^	70 ± 0 ^D,a^	70 ± 0 ^C,a^
	4	0 ± 0 ^D,c^	0 ± 0 ^E,c^	0 ± 0 ^E,c^	0 ± 0 ^E,c^	0 ± 0 ^E,c^	53.3 ± 3.3 ^D,b^	0 ± 0 ^E,c^	60 ± 0 ^E,a^	56.7 ± 3.3 ^D,a,b^
	5	0 ± 0 ^D,d^	0 ± 0 ^E,d^	0 ± 0 ^E,d^	0 ± 0 ^E,d^	0 ± 0 ^E,d^	26.7 ± 3.3 ^E,c^	0 ± 0 ^E,d^	43.3 ± 3.3 ^F,b^	50 ± 0 ^D,a^
*Lolium rigidum* Gaudich.	0	76.7 ± 3.3 ^A,a^	76.7 ± 3.3 ^A,a^	76.7 ± 3.3 ^A,a^	76.7 ± 3.3 ^A,a^	76.7 ± 3.3 ^A,a^	76.7 ± 3.3 ^A,a^	76.7 ± 3.3 ^A,a^	76.7 ± 3.3 ^A,a^	76.7 ± 3.3 ^A,a^
	1	63.3 ± 3.3 ^B,a^	63.3 ± 3.3 ^B,a^	36.7 ± 3.3 ^B,c^	50 ± 0 ^B,b^	53.3 ± 3.3 ^B,a,b^	56.7 ± 3.3 ^B,a,b^	50 ± 0 ^B,b^	60 ± 0 ^B,a,b^	0 ± 0 ^B,d^
	2	53.3 ± 3.3 ^C,a^	36.7 ± 3.3 ^C,c,d^	30 ± 0 ^B,C,d^	0 ± 0 ^C,f^	16.7 ± 3.3 ^C,e^	40 ± 0 C^b,c^	30 ± 0 ^C,d^	40 ± 0 ^C,b,c^	0 ± 0 ^B,f^
	3	0 ± 0 ^D,e^	0 ± 0 ^D,e^	23.3 ± 3.3 ^C,D,c^	0 ± 0 ^C,e^	0 ± 0 ^D,e^	30 ± 0 ^D,b^	10 ± 0 ^D,d^	36.7 ± 3.3 ^C,a^	0 ± 0 ^B,e^
	4	0 ± 0 ^D,c^	0 ± 0 ^D,c^	16.7 ± 3.3 ^D,b^	0 ± 0 ^C,c^	0 ± 0 ^D,c^	0 ± 0 ^E,c^	0 ± 0 ^E,c^	30 ± 0 ^D,a^	0 ± 0 ^B,c^
	5	0 ± 0 ^D,a^	0 ± 0 ^D,a^	0 ± 0 ^E,a^	0 ± 0 ^C,a^	0 ± 0 ^D,a^	0 ± 0 ^E,a^	0 ± 0 ^E,a^	0 ± 0 ^E,a^	0 ± 0 ^B,a^
*Sinapis arvensis* L.	0	93.3 ± 3.3 ^A,a^	93.3 ± 3.3 ^A,a^	93.3 ± 3.3 ^A,a^	93.3 ± 3.3 ^A,a^	93.3 ± 3.3 ^A,a^	93.3 ± 3.3 ^A,a^	93.3 ± 3.3 ^A,a^	93.3 ± 3.3 ^A,a^	93.3 ± 3.3 ^A,a^
	1	0 ± 0 ^B,f^	0 ± 0 ^B,f^	33.3 ± 3.3 ^B,d^	30 ± 0 ^B,d^	50 ± 0 ^B,b^	40 ± 0 ^B,c^	0 ± 0 ^B,f^	63.3 ± 3.3 ^B,d^	50 ± 0 ^B,b^
	2	0 ± 0 ^B,e^	0 ± 0 ^B,e^	16.7 ± 3.3 ^C,c,d^	0 ± 0 ^C,e^	10 ± 0 ^C,d^	20 ± 0 ^C,b^	0 ± 0 ^B,e^	0 ± 0 ^C,e^	50 ± 0 ^B,a^
	3	0 ± 0 ^B,b^	0 ± 0 ^B,b^	0 ± 0 ^D,b^	0 ± 0 ^C,b^	0 ± 0 ^D,b^	0 ± 0 ^D,b^	0 ± 0 ^B,b^	0 ± 0 ^C,b^	30 ± 0 ^C,a^
	4	0 ± 0 ^B,b^	0 ± 0 ^B,b^	0 ± 0 ^D,b^	0 ± 0 ^C,b^	0 ± 0 ^D,b^	0 ± 0 ^D,b^	0 ± 0 ^B,b^	0 ± 0 ^C,b^	30 ± 0 ^C,a^
	5	0 ± 0 ^B,a^	0 ± 0 ^B,a^	0 ± 0 ^D,a^	0 ± 0 ^C,a^	0 ± 0 ^D,a^	0 ± 0 ^D,a^	0 ± 0 ^B,a^	0 ± 0 ^C,a^	20 ± 0 ^D,a^
*Lepidium sativum* L.	0	100 ± 0 ^A,a^	100 ± 0 ^A,a^	100 ± 0 ^A,a^	100 ± 0 ^A,a^	100 ± 0 ^A,a^	100 ± 0 ^A,a^	100 ± 0 ^A,a^	100 ± 0 ^A,a^	100 ± 0 ^A,a^
	1	30 ± 0 ^B,e^	90 ± 0 ^B,a,b^	90 ± 0 ^B,a,b^	70 ± 0 ^A,d^	93.3 ± 3.3 ^B,a^	83.3 ± 3.3 ^B,b,c^	93.3 ± 3.3 ^A,a^	80 ± 0 ^B,c^	90 ± 0 ^B,a,b^
	2	16.7 ± 3.3 ^C,f^	86.7 ± 3.3 ^B,a^	80 ± 0 ^C,a,b^	30 ± 0 ^A,e^	80 ± 0 ^C,a,b^	70 ± 0 ^C,d^	73.3 ± 3.3 ^B,b,c^	60 ± 0 ^C,d^	76.7 ± 3.3 ^C,b,c^
	3	0 ± 0 ^D,f^	66.7 ± 3.3 ^C,a,b^	73.3 ± 3.3 ^D,a^	10 ± 0 ^A,e^	73.3 ± 3.3 ^D,a^	26.7 ± 3.3 ^D,d^	60 ± 0 ^C,b^	50 ± 0 ^D,c^	60 ± 0 ^D,b^
	4	0 ± 0 ^D,d^	46.7 ± 3.3 ^D,b^	60 ± 0 ^E,a^	0 ± 0 ^A,d^	20 ± 0 ^E,a^	0 ± 0 ^E,d^	16.7 ± 3.3 ^D,c^	46.7 ± 3.3 ^D,b^	53.3 ± 3.3 ^D,b^
	5	0 ± 0 ^D,c^	33.3 ± 3.3 ^E,b^	0 ± 0 ^F,c^	0 ± 0 ^A,c^	0 ± 0 ^E,c^	0 ± 0 ^E,c^	0 ± 0 ^E,c^	30 ± 0 ^E,b^	43.3 ± 3.3 ^E,a^
*Raphanus sativus* L.	0	83.3 ± 3.3 ^A,a^	83.3 ± 3.3 ^A,a^	83.3 ± 3.3 ^A,a^	83.3 ± 3.3 ^A,a^	83.3 ± 3.3 ^A,a^	83.3 ± 3.3 ^A,a^	83.3 ± 3.3 ^A,a^	83.3 ± 3.3 ^A,a^	83.3 ± 3.3 ^A,a^
	1	0 ± 0 ^B,f^	30 ± 0 ^B,d^	36.7 ± 3.3 ^B,c,d^	30 ± 0 ^B,d^	26.7 ± 3.3 ^B,d^	50 ± 0 ^B,b^	20 ± 0 ^B,e^	40 ± 0 ^B,c^	60 ± 0 ^B,a^
	2	0 ± 0 ^B,f^	23.3 ± 3.3 ^C,c,d^	30 ± 0 ^B,b,c^	20 ± 0 ^C,d^	20 ± 0 ^C,d^	33.3 ± 3.3 ^C,b^	10 ± 0 ^C,e^	33.3 ± 3.3 ^B,b^	60 ± 0 ^B,a^
	3	0 ± 0 ^B,e^	0 ± 0 ^D,e^	16.7 ± 3.3 ^C,c^	0 ± 0 ^D,e^	10 ± 0 ^D,d^	20 ± 0 ^D,b,c^	0 ± 0 ^D,e^	23.3 ± 3.3 ^C,b^	60 ± 0 ^B,a^
	4	0 ± 0 ^B,b^	0 ± 0 ^D,b^	10 ± 0 ^C,b^	0 ± 0 ^D,b^	0 ± 0 ^E,b^	0 ± 0 ^E,b^	0 ± 0 ^D,b^	20 ± 0 ^C,b^	60 ± 0 ^B,a^
	5	0 ± 0 ^B,c^	0 ± 0 ^D,c^	0 ± 0 ^D,c^	0 ± 0 ^D,c^	0 ± 0 ^E,c^	0 ± 0 ^E,c^	0 ± 0 ^D,c^	13.3 ± 3.3 ^C,b^	50 ± 0 ^C,a^
*Triticum durum* Desf.	0	90 ± 0 ^A,a^	90 ± 0 ^A,a^	90 ± 0 ^A,a^	90 ± 0 ^A,a^	90 ± 0 ^A,a^	90 ± 0 ^A,a^	90 ± 0 ^A,a^	90 ± 0 ^A,a^	90 ± 0 ^A,a^
	1	60 ± 0 ^B,c^	76.7 ± 3.3 ^B,b^	60 ± 0 ^B,c^	33.3 ± 3.3 ^B,d^	80 ± 0 ^B,a,b^	30 ± 0 ^A,d^	20 ± 0 ^B,e^	60 ± 0 ^B,c^	60 ± 0 ^B,c^
	2	53.3 ± 3.3 ^C,c^	50 ± 0 ^C,c^	40 ± 0 ^C,d^	0 ± 0 ^C,f^	70 ± 0 ^C,b^	10 ± 0 ^A,e^	10 ± 0 ^C,e^	50 ± 0 ^C,c^	53.3 ± 3.3 ^C,c^
	3	50 ± 0 ^C,b^	40 ± 0 ^D,c^	30 ± 0 ^D,d^	0 ± 0 ^C,e^	56.7 ± 3.3 ^D,a^	0 ± 0 ^A,e^	0 ± 0 ^D,e^	33.3 ± 3.3 ^D,d^	50 ± 0 ^C,b^
	4	43.3 ± 3.3 ^D,a^	33.3 ± 3.3 ^E,b^	10 ± 0 ^E,d^	0 ± 0 ^C,e^	0 ± 0 ^E,e^	0 ± 0 ^A,e^	0 ± 0 ^D,e^	23.3 ± 3.3 ^E,c^	43.3 ± 3.3 ^D,a^
	5	40 ± 0 ^D,a^	0 ± 0 ^F,b^	0 ± 0 ^F,b^	0 ± 0 ^C,b^	0 ± 0 ^E,b^	0 ± 0 ^A,b^	0 ± 0 ^D,b^	0 ± 0 ^F,b^	40 ± 0 ^D,a^

Means with different capital letters in the same column and for the same tested plant compare the difference between doses according to the Student–Newman–Keuls test at (*p* ≤ 0.05). Means with different lowercase letters in the same line correspond to significant differences according to the Student–Newman–Keuls test at (*p* ≤ 0.05).

**Table 6:** Inhibitory effect of the *Eucalyptus* EOs on root length (cm) of weeds and cultivated crops.

Tested Plants	Doses (μL/mL)	*E. cladocalyx*	*E. microcorys*	*E. resinifera*	*E. saligna*	*E. angulosa*	*E. diversicolor*	*E. ovata*	*E. sargentii*	Glyphosate
*Trifolium campestre* Schreb.	0	5.17 ± 0.04 ^A,a^	5.17 ± 0.04 ^A,a^	5.17 ± 0.04 ^A,a^	5.17 ± 0.04 ^A,a^	5.17 ± 0.04 ^A,a^	5.17 ± 0.04 ^A,a^	5.17 ± 0.04 ^A,a^	5.17 ± 0.04 ^A,a^	5.17 ± 0.04 ^A,a^
	1	1.37 ± 0.02 ^B,f^	1.45 ± 0.01 ^B,f^	3.73 ± 0.04 ^B,b^	2.25 ± 0.08 ^B,d^	2.86 ± 0.02 ^B,c^	4.72 ± 0.09 ^B,a^	2.87 ± 0.07 ^B,c^	3.61 ± 0.03 ^B,b^	0.64 ± 0.01 ^B,g^
	2	0.83 ± 0.02 ^C,g^	0.25 ± 0.06 ^C,j^	2.86 ± 0.04 ^C,b^	0.42 ± 0.02 ^C,i^	1.50 ± 0.02 ^C,f^	3.26 ± 0.05 ^C,a^	1.93 ± 0 ^C,d^	2.09 ± 0.02 ^C,c^	0.59 ± 0.04 ^B,h^
	3	0 ± 0 ^D,h^	0.17 ± 0.03 ^C,g^	0.70 ± 0.03 ^D,e^	0.2 ± 0 ^D,g^	1.02 ± 0 ^D,d^	2.31 ± 0.03 ^D,a^	0.64 ± 0.01 ^D,e,f^	1.51 ± 0.05 ^D,c^	0.6 ± 0.01 ^B,f^
	4	0 ± 0 ^D,e^	**0 ± 0 ^D,e^**	0 ± 0 ^E,e^	0 ± 0 ^E,e^	0 ± 0 ^E,e^	1.58 ± 0.03 ^E,a^	0 ± 0 ^E,e^	0.89 ± 0.04 ^E,c^	0.47 ± 0.01 ^C,d^
	5	0 ± 0 ^D,d^	0 ± 0 ^D,d^	0 ± 0 ^E,d^	0 ± 0 ^E,d^	0 ± 0 ^E,d^	0.26 ± 0.02 ^F,c^	0 ± 0 ^E,d^	0.29 ± 0.01 ^F,c^	0.41 ± 0.01 ^C,b^
*Lolium rigidum* Gaudich.	0	4.66 ± 0.05 ^A,a^	4.66 ± 0.05 ^A,a^	4.66 ± 0.05 ^A,a^	4.66 ± 0.05 ^A,a^	4.66 ± 0.05 ^A,a^	4.66 ± 0.05 ^A,a^	4.66 ± 0.05 ^A,a^	4.66 ± 0.05 ^A,a^	4.66 ± 0.05 ^A,a^
	1	2.87 ± 0.09 ^B,c^	2.48 ± 0.10 ^B,d^	1.86 ± 0.02 ^B,e^	1.67 ± 0.05 ^B,e^	2.63 ± 0.05 ^B,d^	3.98 ± 0.09 ^B,a^	3.38 ± 0.02 ^B,b^	4.16 ± 0.06 ^B,a^	0 ± 0 ^B,f^
	2	1.07 ± 0.04 ^C,d,e^	2.32 ± 0.06 ^B,a^	0.71 ± 0.02 ^C,f^	0 ± 0 ^C,g^	0.87 ± 0.04 ^C,e,f^	1.17 ± 0.01 ^C,d^	1.96 ± 0.03 ^C,b^	1.43 ± 0.08 ^C,c^	0 ± 0 ^B,g^
	3	0 ± 0 ^D,d^	0 ± 0 ^C,d^	0.51 ± 0.02 ^D,c^	0 ± 0 ^C,d^	**0 ± 0 ^D,d^**	0.66 ± 0.02 ^D,b^	0.53 ± 0.03 ^D,c^	0.55 ± 0.06 ^D,c^	0 ± 0 ^B,d^
	4	0 ± 0 ^D,d^	0 ± 0 ^C,d^	0.23 ± 0.03 ^E,c^	0 ± 0 ^C,d^	0 ± 0 ^D,d^	0 ± 0 ^E,d^	0 ± 0 ^E,d^	0.3 ± 0.05 ^E,b^	0 ± 0 ^B,d^
	5	0 ± 0 ^D,b^	0 ± 0 ^C,b^	0 ± 0 ^F,b^	0 ± 0 ^C,b^	0 ± 0 ^D,b^	0 ± 0 ^E,b^	0 ± 0 ^E,b^	0 ± 0 ^F,b^	0 ± 0 ^B,b^
*Sinapis arvensis* L.	0	3.74 ± 0.08 ^A,a^	3.74 ± 0.08 ^A,a^	3.74 ± 0.08 ^A,a^	3.74 ± 0.08 ^A,a^	3.74 ± 0.08 ^A,a^	3.74 ± 0.08 ^A,a^	3.74 ± 0.08 ^A,a^	3.74 ± 0.08 ^A,a^	3.74 ± 0.08 ^A,a^
	1	0 ± 0 ^B,g^	0 ± 0 ^B,g^	2.66 ± 0.08 ^B,b^	1.70 ± 0.05 ^B,c^	3.48 ± 0.20 ^A,a^	1.25 ± 0.03 ^B,d^	0 ± 0 ^B,g^	1.74 ± 0.06 ^B,c^	0.5 ± 0 ^B,f^
	2	0 ± 0 ^B,d^	0 ± 0 ^B,d^	1.06 ± 0.07 ^C,b^	0 ± 0 ^C,d^	1.73 ± 0.14 ^B,a^	0.65 ± 0.05 ^C,c^	0 ± 0 ^B,d^	0 ± 0 ^C,d^	0.5 ± 0 ^B,c^
	3	0 ± 0 ^B,b^	0 ± 0 ^B,b^	0.48 ± 0.06 ^D,a^	0 ± 0 ^C,b^	0 ± 0 ^C,b^	0 ± 0 ^D,b^	0 ± 0 ^B,b^	0 ± 0 ^C,b^	0.5 ± 0 ^B,a^
	4	0 ± 0 ^B,b^	0 ± 0 ^B,b^	0 ± 0 ^E,b^	0 ± 0 ^C,b^	0 ± 0 ^C,b^	0 ± 0 ^D,b^	0 ± 0 ^B,b^	0 ± 0 ^C,b^	0.5 ± 0 ^B,a^
	5	0 ± 0 ^B,b^	0 ± 0 ^B,b^	0 ± 0 ^E,b^	0 ± 0 ^C,b^	0 ± 0 ^C,b^	0 ± 0 ^D,b^	0 ± 0 ^B,b^	0 ± 0 ^C,b^	0.5 ± 0 ^B,a^
*Lepidium sativum* L.	0	6.52 ± 0.05 ^A,a^	6.52 ± 0.05 ^A,a^	6.52 ± 0.05 ^A,a^	6.52 ± 0.05 ^A,a^	6.52 ± 0.05 ^A,a^	6.52 ± 0.05 ^A,a^	6.52 ± 0.05 ^A,a^	6.52 ± 0.05 ^A,a^	6.52 ± 0.05 ^A,a^
	1	2.30 ± 0.12 ^B,f^	2.98 ± 0.06 ^B,e^	4.24 ± 0.03 ^B,c^	1.07 ± 0.02 ^B,h^	4.44 ± 0.06 ^B,b^	3.40 ± 0.07 ^B,d^	5.58 ± 0.10 ^B,a^	2.07 ± 0.02 ^B,g^	0.72 ± 0.02 ^B,i^
	2	0.63 ± 0.04 ^C,f^	0.79 ± 0.01 ^C,f^	3.60 ± 0.06 ^C,b^	0.34 ± 0.01 ^C,g^	3.64 ± 0.03 ^C,b^	2.74 ± 0.06 ^C,d^	2.89 ± 0.05 ^C,c^	1.61 ± 0.03 ^C,e^	0.66 ± 0.02 ^B,f^
	3	0 ± 0 ^D,i^	0.34 ± 0.01 ^D,g^	2.70 ± 0.12 ^D,c^	0.20 ± 0.00 ^D,h^	3.18 ± 0.01 ^D,a^	0.54 ± 0.04 ^D,f^	1.75 ± 0.07 ^D,d^	1.41 ± 0.02 ^D,e^	0.58 ± 0.01 ^C,f^
	4	0 ± 0 ^D,f^	0.22 ± 0.01 ^E,e^	1.43 ± 0.07 ^E,a^	0 ± 0 ^E,f^	0 ± 0 ^E,f^	0 ± 0 ^E,f^	0.85 ± 0.03 ^E,c^	0.96 ± 0.02 ^E,b^	0.42 ± 0.01 ^D,d^
	5	0 ± 0 ^D,e^	0.12 ± 0.01 ^F,d^	0 ± 0 ^F,e^	0 ± 0 ^E,e^	0 ± 0 ^E,e^	0 ± 0 ^E,e^	0 ± 0 ^F,e^	0.75 ± 0.02 ^F,b^	0.35 ± 0.04 ^D,c^
*Raphanus sativus* L.	0	8.67 ± 0.11 ^A,a^	8.67 ± 0.11 ^A,a^	8.67 ± 0.11 ^A,a^	8.67 ± 0.11 ^A,a^	8.67 ± 0.11 ^A,a^	8.67 ± 0.11 ^A,a^	8.67 ± 0.11 ^A,a^	8.67 ± 0.11 ^A,a^	8.67 ± 0.11 ^A,a^
	1	0 ± 0 ^B,i^	4.43 ± 0.04 ^B,b^	0 ± 0 ^B,i^	1.07 ± 0.03 ^B,g^	5.50 ± 0 ^B,a^	1.43 ± 0.04 ^B,f^	3.58 ± 0.08 ^B,d^	1.89 ± 0.05 ^B,e^	0.5 ± 0 ^B,h^
	2	0 ± 0 ^B,g^	2.75 ± 0.06 ^C,a^	0 ± 0 ^B,g^	0.20 ± 0.00 ^C,f^	2.60 ± 0.08 ^C,b^	1.12 ± 0.04 ^C,c^	0.50 ± 0 ^C,e^	0.73 ± 0.03 ^C,d^	0.5 ± 0 ^B,e^
	3	0 ± 0 ^B,c^	0 ± 0 ^D,c^	0 ± 0 ^B,c^	0 ± 0 ^D,c^	1.03 ± 0.03 ^D,a^	0.47 ± 0.04 ^D,b^	0 ± 0 ^D,c^	0.52 ± 0.02 ^D,b^	0.5 ± 0 ^B,b^
	4	0 ± 0 ^B,c^	0 ± 0 ^D,c^	0 ± 0 ^B,c^	0 ± 0 ^D,c^	0 ± 0 ^E,c^	0 ± 0 ^E,c^	0 ± 0 ^D,c^	0.28 ± 0.02 ^E,b^	0.5 ± 0 ^B,a^
	5	0 ± 0 ^B,c^	0 ± 0 ^D,c^	0 ± 0 ^B,c^	0 ± 0 ^D,c^	0 ± 0 ^E,c^	0 ± 0 ^E,c^	0 ± 0 ^D,c^	0.13 ± 0.02 ^E,b^	0.5 ± 0 ^B,a^
*Triticum durum* Desf.	0	15.42 ± 0.04 ^A,a^	15.42 ± 0.04 ^A,a^	15.42 ± 0.04 ^A,a^	15.42 ± 0.04 ^A,a^	15.42 ± 0.04 ^A,a^	15.42 ± 0.04 ^A,a^	15.42 ± 0.04 ^A,a^	15.42 ± 0.04 ^A,a^	15.42 ± 0.04 ^A,a^
	1	0.50 ± 0 ^B,g^	9.80 ± 0.15 ^B,b^	10.89 ± 0.08 ^B,a^	7.83 ± 0.60 ^B,c^	6.71 ± 0.17 ^B,d^	3.29 ± 0.01 ^B,f^	5.63 ± 0.07 ^B,e^	11.37 ± 0.16 ^B,a^	0.35 ± 0 ^B,g^
	2	0 ± 0 ^C,h^	5.63 ± 0.12 ^C,c^	7.07 ± 0.02 ^C,a^	4.83 ± 0.17 C^d,e^	4.70 ± 0.10 ^C,e^	1.00 ± 0 ^C,g^	2.90 ± 0.06 ^C,f^	5.01 ± 0.04 ^C,d^	0.20 ± 0 ^C,h^
	3	0 ± 0 ^C,g^	2.98 ± 0.05 ^D,c^	5.58 ± 0.06 ^D,a^	2 ± 0 ^D,e^	2.30 ± 0.08 ^D,d^	0 ± 0 ^D,g^	0 ± 0 ^D,g^	3.86 ± 0.05 ^D,b^	0.20 ± 0 ^C,f^
	4	0 ± 0 ^C,f^	1.87 ± 0.02 ^E,b^	2.00 ± 0 ^E,a^	0 ± 0 ^E,f^	0 ± 0 ^E,f^	0 ± 0 ^D,f^	0 ± 0 ^D,f^	0.88 ± 0.01 ^E,d^	0.20 ± 0 ^C,e^
	5	0 ± 0 ^C,b^	0 ± 0 ^F,b^	0 ± 0 ^F,b^	0 ± 0 ^E,b^	0 ± 0 ^E,b^	0 ± 0 ^D,b^	0 ± 0 ^D,b^	0 ± 0 ^F,b^	0.20 ± 0 ^C,a^

Means with different capital letters in the same column and for the same tested plant compare the difference between doses according to the Student–Newman–Keuls test at (*p* ≤ 0.05). Means with different lowercase letters in the same line correspond to significant differences according to the Student–Newman–Keuls test at (*p* ≤ 0.05).

**Table 7:** Inhibitory effect of the *Eucalyptus* EOs on shoot length (cm) of weeds and cultivated crops.

Tested Plants	Doses (μL/mL)	*Eucalyptus* *cladocalyx*	*Eucalyptus* *microcorys*	*Eucalyptus* *resinifera*	*Eucalyptus* *saligna*	*Eucalyptus* *angulosa*	*Eucalyptus* *diversicolor*	*Eucalyptus* *ovata*	*Eucalyptus* *sargentii*	Glyphosate
*Trifolium campestre* Schreb.	0	1.97 ± 0.01 ^A,a^	1.97 ± 0.01 ^A,a^	1.97 ± 0.01 ^A,a^	1.97 ± 0.01 ^A,a^	1.97 ± 0.01 ^A,a^	1.97 ± 0.01 ^A,a^	1.97 ± 0.01 ^A,a^	1.97 ± 0.01 ^A,a^	1.97 ± 0.01 ^A,a^
	1	1.01 ± 0.01 ^B,c^	0.54 ± 0.02 ^B,g^	1.37 ± 0.01 ^B,a^	0.70 ± 0.01 ^B,f^	1.02 ± 0.01 ^B,c^	0.88 ± 0.02 ^B,d^	0.82 ± 0.01 ^B,e^	0.78 ± 0.02 ^B,e^	0.23 ± 0.01 ^B,h^
	2	0.72 ± 0.03 ^C,b^	0.22 ± 0 ^C,f^	1.06 ± 0.03 ^C,a^	0.50 ± 0 ^C,e^	0.65 ± 0.02 ^C,c^	0.60 ± 0.02 ^C,c,d^	0.54 ± 0.01 ^C,d,e^	0.55 ± 0.01 ^C,d,e^	0.18 ± 0.01 ^C,f^
	3	0 ± 0 ^D,f^	0.10 ± 0 ^D,e^	0.45 ± 0.01 ^D,b^	0.32 ± 0.02 ^D,c^	0.37 ± 0.00 ^D,c^	0.49 ± 0 ^D,b^	0.31 ± 0.01 ^D,c^	0.47 ± 0.01 ^D,b^	0.18 ± 0.01 ^C,d^
	4	0 ± 0 ^D,d^	0 ± 0 ^E,d^	0 ± 0 ^E,d^	0 ± 0 ^E,d^	0 ± 0 ^E,d^	0.34 ± 0.02 ^E,b^	0 ± 0 ^E,d^	0.35 ± 0.01 ^E,b^	0.15 ± 0.01 ^C,c^
	5	0 ± 0 ^D,c^	0 ± 0 ^E,c^	0 ± 0 ^E,c^	0 ± 0 ^E,b,c^	0 ± 0 ^E,c^	0.19 ± 0.03 ^F,b^	0 ± 0 ^E,c^	0.16 ± 0.02 ^F,c^	0.15 ± 0.01 ^C,b^
*Lolium rigidum* Gaudich.	0	4.04 ± 0 ^A,a^	4.04 ± 0 ^A,a^	4.04 ± 0 ^A,a^	4.04 ± 0 ^A,a^	4.04 ± 0 ^A,a^	4.04 ± 0 ^A,a^	4.04 ± 0 ^A,a^	4.04 ± 0 ^A,a^	4.04 ± 0 ^A,a^
	1	2.60 ± 0.09 ^B,c^	2.21 ± 0.03 ^B,d^	1.65 ± 0.02 ^B,e^	1.39 ± 0.08 ^B,f^	2.47 ± 0.02 ^B,c,d^	3.54 ± 0.12 ^B,a^	3.14 ± 0.02 ^B,b^	3.28 ± 0.10 ^B,b^	0 ± 0 ^B,g^
	2	1.18 ± 0.05 ^C,e^	1.50 ± 0.03 ^C,d^	0.47 ± 0.01 ^C,g^	0 ± 0 ^C,h^	0.73 ± 0.06 ^C,f^	2.39 ± 0.08 ^C,a^	2.08 ± 0.01 ^C,b^	2.14 ± 0.03 ^C,b^	0 ± 0 ^B,h^
	3	0 ± 0 ^D,e^	0 ± 0 ^D,e^	0.29 ± 0.01 ^D,d^	0 ± 0 ^C,e^	0 ± 0 ^D,e^	0.72 ± 0.06 ^D,c^	0.93 ± 0.03 ^D,b^	1.10 ± 0.09 ^D,a^	0 ± 0 ^B,g^
	4	0 ± 0 ^D,b^	0 ± 0 ^D,b^	0.12 ± 0.02 ^E,b^	0 ± 0 ^C,b^	0 ± 0 ^D,b^	0 ± 0 ^E,b^	0 ± 0 ^E,b^	0.57 ± 0.10 ^E,a^	0 ± 0 ^B,b^
	5	0 ± 0 ^D,b^	0 ± 0 ^D,b^	0 ± 0 ^F,b^	0 ± 0 ^C,b^	0 ± 0 ^D,b^	0 ± 0 ^E,b^	0 ± 0 ^E,b^	0 ± 0 ^F,b^	0 ± 0 ^B,b^
*Sinapis arvensis* L.	0	2.77 ± 0.03 ^A,a^	2.77 ± 0.03 ^A,a^	2.77 ± 0.03 ^A,a^	2.77 ± 0.03 ^A,a^	2.77 ± 0.03 ^A,a^	2.77 ± 0.03 ^A,a^	2.77 ± 0.03 ^A,a^	2.77 ± 0.03 ^A,a^	2.77 ± 0.03 ^A,a^
	1	0 ± 0 ^B,g^	0 ± 0 ^B,g^	0.92 ± 0.04 ^B,d^	0.59 ± 0.01 ^B,e^	1.80 ± 0.02 ^B,c^	1.89 ± 0.05 ^B,b^	0 ± 0 ^B,g^	2.17 ± 0.03 ^B,a^	0.50 ± 0 ^B,f^
	2	0 ± 0 ^B,d^	0 ± 0 ^B,d^	0.55 ± 0.03 ^C,b^	0 ± 0 ^C,d^	0.87 ± 0.03 ^C,a^	0.08 ± 0.02 ^C,d^	0 ± 0 ^B,d^	0 ± 0 ^C,d^	0.50 ± 0 ^B,b^
	3	0 ± 0 ^B,b^	0 ± 0 ^B,b^	0 ± 0 ^D,b^	0 ± 0 ^C,b^	0 ± 0 ^D,b^	0 ± 0 ^C,b^	0 ± 0 ^B,b^	0 ± 0 ^C,b^	0.50 ± 0 ^B,a^
	4	0 ± 0 ^B,b^	0 ± 0 ^B,b^	0 ± 0 ^D,b^	0 ± 0 ^C,b^	0 ± 0 ^D,b^	0 ± 0 ^C,b^	0 ± 0 ^B,b^	0 ± 0 ^C,b^	0.50 ± 0 ^B,a^
	5	0 ± 0 ^B,b^	0 ± 0 ^B,b^	0 ± 0 ^D,b^	0 ± 0 ^C,b^	0 ± 0 ^D,b^	0 ± 0 ^C,b^	0 ± 0 ^B,b^	0 ± 0 ^C,b^	0.50 ± 0 ^B,a^
*Lepidium sativum* L.	0	1.42 ± 0.02 ^A,a^	1.42 ± 0.02 ^A,a^	1.42 ± 0.02 ^A,a^	1.42 ± 0.02 ^A,a^	1.42 ± 0.02 ^A,a^	1.42 ± 0.02 ^A,a^	1.42 ± 0.02 ^A,a^	1.42 ± 0.02 ^A,a^	1.42 ± 0.02 ^A,a^
	1	0.96 ± 0.03 ^B,a^	0.77 ± 0.04 ^B,b^	0.74 ± 0.07 ^B,b^	0.54 ± 0 ^B,c^	0.68 ± 0.06 ^B,b^	0.96 ± 0.01 ^B,a^	0.87 ± 0.01 ^B,a^	0.90 ± 0.01 ^B,a^	0.31 ± 0.01 ^B,d^
	2	0.67 ± 0.02 ^C,b^	0.64 ± 0.02 ^C,b^	0.67 ± 0.04 ^B,b^	0.31 ± 0.01 ^C,d^	0.52 ± 0.01 ^C,c^	0.78 ± 0.03 ^C,a^	0.51 ± 0.01 ^C,c^	0.75 ± 0.02 ^C,a^	0.25 ± 0 ^C,d^
	3	0 ± 0 ^D,e^	0.55 ± 0.02 ^D,a^	0.55 ± 0.02 ^C,a^	0.20 ± 0 ^D,d^	0 ± 0 ^D,e^	0.49 ± 0.02 ^D,b^	0.37 ± 0.02 ^D,c^	0.47 ± 0.01 ^D,b^	0.21 ± 0.01 ^D,d^
	4	0 ± 0 ^D,d^	0.49 ± 0.01 ^D,a^	0.52 ± 0.04 ^C,a^	0 ± 0 ^E,d^	0 ± 0 ^D,d^	0 ± 0 ^E,d^	0.17 ± 0.03 ^E,c^	0.35 ± 0.02 ^E,b^	0.15 ± 0 ^E,c^
	5	0 ± 0 ^D,d^	0 ± 0 ^E,d^	0 ± 0 ^D,d^	0 ± 0 ^E,d^	0 ± 0 ^D,d^	0 ± 0 ^E,d^	0 ± 0 ^F,d^	0.25 ± 0.02 ^F,b^	0.10 ± 0 ^F,c^
*Raphanus sativus* L.	0	2.56 ± 0.04 ^A,a^	2.56 ± 0.04 ^A,a^	2.56 ± 0.04 ^A,a^	2.56 ± 0.04 ^A,a^	2.56 ± 0.04 ^A,a^	2.56 ± 0.04 ^A,a^	2.56 ± 0.04 ^A,a^	2.56 ± 0.04 ^A,a^	2.56 ± 0.04 ^A,a^
	1	0 ± 0 ^B,g^	2.43 ± 0.04 ^B,a^	0 ± 0 ^B,g^	0.49 ± 0.01 ^B,f^	1.33 ± 0.03 ^B,d^	1.19 ± 0.02 ^B,e^	1.53 ± 0.02 ^B,c^	2.08 ± 0.04 ^B,b^	0.50 ± 0 ^B,f^
	2	0 ± 0 ^B,h^	2.09 ± 0.05 ^C,a^	0 ± 0 ^B,h^	0.17 ± 0.02 ^C,g^	0.93 ± 0.02 ^C,d^	0.62 ± 0.02 ^C,e^	0.97 ± 0.03 ^C,d^	1.88 ± 0.05 ^C,b^	0.50 ± 0 ^B,f^
	3	0 ± 0 ^B,d^	0 ± 0 ^D,d^	0 ± 0 ^B,d^	0 ± 0 ^D,d^	0.37 ± 0.03 ^D,c^	0.45 ± 0.03 ^D,b^	0 ± 0 ^D,d^	1.36 ± 0.05 ^D,a^	0.50 ± 0 ^B,b^
	4	0 ± 0 ^B,c^	0 ± 0 ^D,c^	0 ± 0 ^B,c^	0 ± 0 ^D,c^	0 ± 0 ^E,c^	0 ± 0 ^E,c^	0 ± 0 ^D,c^	0.23 ± 0.02 ^E,b^	0.50 ± 0 ^B,a^
	5	0 ± 0 ^B,b^	0 ± 0 ^D,b^	0 ± 0 ^B,b^	0 ± 0 ^D,b^	0 ± 0 ^E,b^	0 ± 0 ^E,b^	0 ± 0 ^D,b^	0 ± 0 ^F,b^	0.50 ± 0 ^B,b^
*Triticum durum* Desf.	0	11.47 ± 0.02 ^A,a^	11.47 ± 0.02 ^A,a^	11.47 ± 0.02 ^A,a^	11.47 ± 0.02 ^A,a^	11.47 ± 0.02 ^A,a^	11.47 ± 0.02 ^A,a^	11.47 ± 0.02 ^A,a^	11.47 ± 0.02 ^A,a^	11.47 ± 0.02 ^A,a^
	1	0.50 ± 0 ^B,h^	7.21 ± 0.22 ^B,d^	8.86 ± 0.04 ^B,c^	10.13 ± 0.19 ^B,a,b^	5.44 ± 0.07 ^B,f^	9.84 ± 0.10 ^B,b^	5.90 ± 0.06 ^B,e^	10.5 ± 0.25 ^B,a^	0.44 ± 0.01 ^B,h^
	2	0 ± 0 ^C,i^	2.84 ± 0.03 ^C,g^	5.36 ± 0.03 ^C,b^	4.90 ± 0.06 ^C,c^	3.48 ± 0.04 ^C,e^	7.10 ± 0.06 ^C,a^	3.97 ± 0.09 ^C,d^	7.05 ± 0.04 ^C,a^	0.20 ± 0 ^C,h^
	3	0 ± 0 ^C,g^	0.53 ± 0 ^D,e^	3.51 ± 0.04 ^D,b^	0.87 ± 0.09 ^D,d^	0.62 ± 0.04 ^D,e^	0 ± 0 ^D,g^	0 ± 0 ^D,g^	3.99 ± 0.03 ^D,a^	0.20 ± 0 ^C,f^
	4	0 ± 0 ^C,f^	0.52 ± 0.04 ^D,c^	1.50 ± 0 ^E,a^	0 ± 0 ^E,f^	0 ± 0 ^E,f^	0 ± 0 ^D,f^	0 ± 0 ^D,f^	0.60 ± 0.03 ^E,b^	0.20 ± 0 ^C,e^
	5	0 ± 0 ^C,b^	0 ± 0 ^E,b^	0 ± 0 ^F,b^	0 ± 0 ^E,b^	0 ± 0 ^E,b^	0 ± 0 ^D,b^	0 ± 0 ^D,b^	0 ± 0 ^F,b^	0.20 ± 0 ^C,a^

Means with different capital letters in the same column and for the same tested plant compare the difference between doses according to the Student–Newman–Keuls test at (*p* ≤ 0.05). Means with different lowercase letters in the same line correspond to significant differences according to the Student–Newman–Keuls test at (*p* ≤ 0.05).

**Table 8:** *Eucalyptus* species, date and sites of harvest.

Species	Preserved Specimen Code	Date of Harvest	Harvest Site	Bioclimatic Stage
*E. ovata* Labill.	EOV211	December 2021	Arboretum of Zerniza (Region of Sejnene)	Humid lower biocli-matic
*E. resinifera* Sm.	ERE212			
*E. angulosa* Schauer	EAN213			
*E. microcorys* F.Muell.	EMI214			
*E. diversicolor* F.Muell.	EDI227	September 2022	Arboretum of Zerniza (Region of Sejnene)	Humid lower bioclimatic
*E. saligna* Sm.	*ESA222*			
*E. cladocalyx* F.Muell.	ECL224			
*E. sargentii* Maiden	ESAR218	January 2021	Arboretum of Hanya (Governorate of Sousse)	Semiarid bioclimate

## Data Availability

All data are available in the manuscript file.
